# Body mass index and extent of MRI-detected inflammation: opposite effects in rheumatoid arthritis versus other arthritides and asymptomatic persons

**DOI:** 10.1186/s13075-016-1146-3

**Published:** 2016-10-22

**Authors:** Lukas Mangnus, Wouter P. Nieuwenhuis, Hanna W. van Steenbergen, Tom W. J. Huizinga, Monique Reijnierse, Annette H. M. van der Helm-van Mil

**Affiliations:** 1 Department of Rheumatology, Leiden University Medical Center, P.O. Box 9600, Leiden, 2300 RC The Netherlands; 2 Department of Radiology, Leiden University Medical Center, Leiden, The Netherlands

**Keywords:** Rheumatoid arthritis, Body mass index, Magnetic resonance imaging, Inflammation, Asymptomatic volunteers

## Abstract

**Background:**

In the population a high body mass index (BMI) has been associated with slightly increased inflammatory markers. Within rheumatoid arthritis (RA), however, a high BMI has been associated with less radiographic progression; this phenomenon is unexplained. We hypothesized that the phenomenon is caused by an inverse relationship between BMI and inflammation in hand and foot joints with RA. To explore this hypothesis, local inflammation was measured using magnetic resonance imaging (MRI) in early arthritis patients presenting with RA or other arthritides and in asymptomatic volunteers.

**Methods:**

A total of 195 RA patients, 159 patients with other inflammatory arthritides included in the Leiden Early Arthritis Clinic, and 193 asymptomatic volunteers underwent a unilateral contrast-enhanced 1.5 T MRI scan of metacarpophalangeal, wrist, and metatarsophalangeal joints. Each MRI scan was scored by two readers on synovitis, bone marrow edema (BME), and tenosynovitis; the sum yielded the total MRI inflammation score. Linear regression on log-transformed MRI data was used.

**Results:**

A higher BMI was associated with higher MRI inflammation scores in arthritides other than RA (β = 1.082, *p* < 0.001) and in asymptomatic volunteers (β = 1.029, *p* = 0.040), whereas it was associated with lower MRI inflammation scores in RA (β = 0.97, *p* = 0.005). Evaluating the different types of inflammation, a higher BMI was associated with higher synovitis, BME, and tenosynovitis scores in arthritides other than RA (respectively β = 1.084, *p* < 0.001, β = 1.021, *p* = 0.24, and β = 1.054, *p* = 0.003), but with lower synovitis and BME scores in RA (respectively β = 0.98, *p* = 0.047 and β = 0.95, *p* = 0.002).

**Conclusions:**

Increased BMI is correlated with less severe MRI-detected synovitis and BME in RA. This might explain the paradox in RA where obesity correlates with less severe radiographic progression.

**Electronic supplementary material:**

The online version of this article (doi:10.1186/s13075-016-1146-3) contains supplementary material, which is available to authorized users.

## Background

The prevalence of obesity is increasing worldwide. It has become evident that adipose tissue is an active organ, producing proinflammatory cytokines and adipocytokines. A population-based study has shown that a high body mass index (BMI) is associated with increased inflammatory markers, such as C-reactive protein (CRP) [1]. Obesity is also associated with an increased risk for several diseases, among which is cardiovascular disease, in which low-grade inflammation is part of the pathogenesis. Furthermore, recent data have revealed that obesity is also associated with an increased risk for rheumatoid arthritis (RA) [2].

Interestingly, however, although obese persons have a higher risk to develop RA, the presence of obesity within RA has been shown advantageous. Several studies have observed and replicated that a higher BMI is associated with less severe radiographic joint progression in RA [3–6]. The mechanisms underlying this observation are unknown. Data from a recent clinical trial in RA, evaluating drug efficacy with magnetic resonance imaging (MRI) to measure the disease outcome, suggested that patients with a higher BMI have less severe bone marrow edema (BME) on MRI [3]. BME is strongly associated with erosive progression [7], which may explain the finding of BMI and radiographic progression in RA. Together these observations prompted us to study the association between BMI and MRI-detected joint inflammation in more detail.

This cross-sectional study investigated the association between BMI and inflammation in hand and foot joints detected by MRI at disease presentation; we hypothesized that a higher BMI is associated with less severe local inflammation in RA. Because an advantageous effect of BMI has only been observed thus far in RA, we also evaluated the association between BMI and MRI-detected inflammation differed in RA patients compared with patients with other inflammatory arthritides or with asymptomatic volunteers.

## Methods

### Participants

Three groups were studied. Firstly, RA patients who were consecutively included in the Leiden Early Arthritis Clinic (EAC) cohort between August 2010 and October 2014. Secondly, early arthritis patients with other inflammatory diagnoses who were included in the same cohort in the same time period. Thirdly, asymptomatic volunteers who were recruited from the general population.

The EAC cohort is an inception cohort of early arthritis patients presenting with clinically detected arthritis of ≥ 1 joint and symptom duration < 2 years [8]. At baseline, questionnaires were filled, physical examination was performed (including weight and height), blood samples were obtained, and MRI was performed. RA was defined as fulfilling the 1987 ACR criteria during the first year of follow-up.

The asymptomatic volunteers were recruited between November 2013 and December 2014 [9]. Volunteers were recruited via advertisements in local newspapers and websites. The volunteers had no history of RA or other inflammatory rheumatic diseases, no joint symptoms during the last month, and no clinically detectable arthritis at physical examination. Participants received a voucher of €20 to compensate for their time and travel costs and did not receive a report of the MRI results. Therefore, volunteers had no/limited benefit from participating.

### MRI protocol and scoring

On an ONI-MSK-extreme 1.5 T extremity MRI machine (General Electric, WI, USA), imaging was performed of the unilateral metacarpophalangeal (MCP) 2–5 joints, wrist joints, and metatarsophalangeal (MTP) 1–5 joints. In patients the most painful side was scanned or, in case of equally severe symptoms on both sides, the dominant side. In asymptomatic volunteers, the dominant side was scanned. According to the protocol, the MRI scan was performed before the 2-week visit in which patients receive their diagnosis, and the median time between the first visit and the MRI scan was 8 days. Furthermore, patients were asked to stop NSAIDS 24 hours prior to the MRI scan. The following sequences were acquired: T1 fast-spin echo (T1), T2-weighted fat saturated (T2), and, after intravenous contrast administration (gadoteric acid, 0.1 mmol/kg; Guerbet, Paris, France), T1 fast-spin echo with fat saturation (T1 Gd). A detailed scan protocol is provided in Additional file 1. Scoring was carried out according to the RAMRIS for synovitis and BME in the MCP, wrist, and MTP joints and according to Haavardsholm et al. for tenosynovitis in the MCP and wrist [10, 11]. The total MRI inflammation score was calculated by summing the synovitis, BME, and tenosynovitis scores. MRI scoring of the arthritis patients was done independently by two trained readers (WPN and ECN) and the asymptomatic volunteers were scored independently by two trained readers (HWvS and LM). All readers were trained before the start of this project. Readers were blinded for any clinical data. MRI images of asymptomatic volunteers were blinded and mixed with MRI images of RA patients and patients with arthralgia without clinical synovitis (*n* = 99), to exclude observer bias scoring introduced by knowledge that persons had no symptoms. The within-reader intraclass correlation coefficients (ICC) for the readers who scored the arthritis patients were 0.98 and 0.93, and for the readers who scored the asymptomatic volunteers these were 0.98 and 0.99. The between-reader ICC of the four readers were all above 0.91 (Additional file 2). The mean scores of two readers were used for the analyses.

### Analyses

Associations between MRI-detected inflammation and BMI were assessed using univariable and multivariable linear regression analyses. The MRI inflammation scores were log_10_-transformed (log_10_(score + 1)) to approximate a normal distribution. Thereafter, BMI was divided into three categories according to the World Health Organization (WHO) definition: low-normal weight (< 25 kg/m^2^), overweight (≥ 25 to < 30 kg/m^2^), and obese (≥ 30 kg/m^2^). The Kruskal–Wallis test, Mann–Whitney *U* test, linear regression models, and Spearman rank correlation coefficient were used as appropriate. SPSS V23.0.0 was used.

In RA patients treated in a trial, an association between BMI and BME was observed [3]. We sought to compare our findings in an unselected RA population at disease presentation with these results. In order to do so, we performed a multivariable ordinal logistic regression model in which BME was categorized into quintiles, similar to that done in the trial [3].

## Results

### Participants

In total, 202 RA patients and 170 early arthritis patients with other inflammatory diagnoses were consecutively included in the Leiden EAC. Five RA patients and eight other arthritis patients had no information on height or weight and, respectively, two and three patients had an MRI scan without contrast enhancement and were excluded. Therefore, 195 RA patients and 159 arthritis patients with other inflammatory diagnoses were evaluated. In addition, 196 asymptomatic volunteers were recruited, as already described, three of whom did not receive an MRI scan due to personal problems (*n* = 1), vasovagal collapse at intravenous puncture (*n* = 1), and anxiety (*n* = 1).

Baseline characteristics of all participants are presented in Table 1. Seventy-nine (41 %) RA patients were overweight (BMI > 25 to < 30 kg/m^2^) and 41 (21 %) were obese (≥ 30 kg/m^2^); in other arthritis patients these percentages were respectively 44 % (*n* = 70) and 14 % (*n* = 23), and in asymptomatic volunteers these percentages were 32 % (*n* = 61) and 9 % (*n* = 17) respectively.

**Table 1:** Characteristics of early rheumatoid arthritis patients, early arthritis patients with other arthritides, and asymptomatic volunteers

	Rheumatoid arthritis (*n* = 195)	Arthritis patients with other arthritides (*n* = 159)	Asymptomatic volunteers (*n* = 193)
Female, *n* (%)	119 (66)	80 (52)	136 (70)
Age, mean (SD)	55.9 (14.6)	54.3 (17.2)	50.7 (26.4)
Symptom duration (months), median (IQR)	3.2 (1.8–6.8)	3.0 (1.0–6.2)	–
Current smokers, *n* (%)	38 (24)	29 (23)	17 (9)
BMI (kg/m^2^), median (IQR)	26.4 (23.7–29.4)	25.5 (22.9–27.9)	24.1 (22.3–26.3)
WHO BMI classification, *n* (%)			
Low-normal weight (BMI 18.5–24.9 kg/m^2^) (%)	75 (38)	66 (42)	115 (60)
Overweight (BMI 25.0–29.9 kg/m^2^) (%)	79 (41)	70 (44)	61 (32)
Obesity (BMI ≥ 30 kg/m^2^) (%)	41 (21)	23 (14)	17 (9)
CRP (mg/L), median (IQR)	9.8 (3.7–23.0)	4.0 (3.0–15.1)	NA
ACPA positivity, *n* (%)	107 (55)	6 (4)	NA
RF positivity, *n* (%)	120 (62)	27 (17)	NA

Three (2 %) RA patients had a low weight (BMI <18.5 kg/m^2^), no patients with other arthritides had a low weight, and two (1 %) asymptomatic volunteers had a low weight

Gender was missing in 11 RA patients; within early arthritis patients with other arthritides, gender, ACPA positivity and RF positivity was missing in respectively six, two, and three patients

*NA* not assessed, *WHO* World Health Organization, *BMI* body mass index, *CRP* C-reactive protein, *ACPA* anti-citrullinated protein antibody, *RF* rheumatoid factor, *RA* rheumatoid arthritis

### BMI and MRI-detected inflammation

The median MRI inflammation score in RA patients was 14.5 (IQR = 7.0–26.5), in other arthritis patients the median was 6.0 (IQR = 2.0–15.0), and in asymptomatic volunteers the median was 2.0 (IQR = 0.5–4.5, *p* < 0.001), showing that RA patients had the highest MRI inflammation scores.

**Fig. 1:**
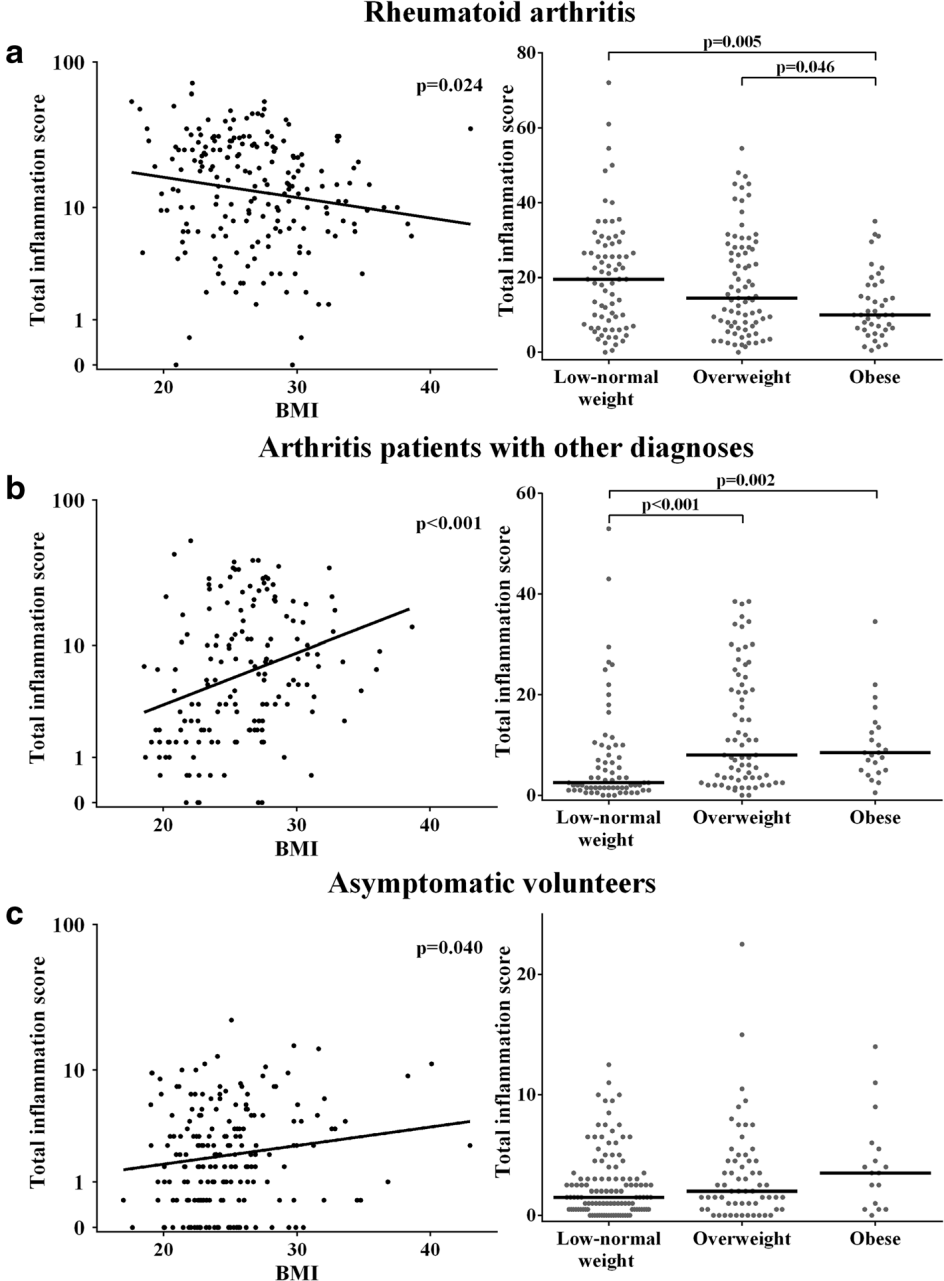
Association between BMI (both when presented on a continuous scale and when categorized) and MRI-detected inflammation is different in early RA patients (**a**) compared with early arthritis patients with other diagnoses (**b**) and asymptomatic volunteers (**c**). Total inflammation scores were log-transformed for regressions. Regression coefficients presented are back-transformed (10^β^ and 10^95 % CI^). In RA patients the back-transformed regression coefficient is 0.97 (95 % CI 0.94–1.00, **a** *left*), in arthritis patients with other diagnoses the coefficient is 1.082 (95 % CI 1.041–1.13, **b** *left*), and in asymptomatic volunteers it was 1.029 (95 % CI 1.001–1.057, **c** *left*). *Horizontal lines* (*right*) represent median values. *BMI* body mass index

In RA patients, a higher BMI was associated with lower MRI inflammation scores (β = 0.97, *p* = 0.024). A β value of 0.97 indicates that for every point increase in BMI there is a 0.97-fold increase in MRI inflammation score; thus higher BMI was associated with less severe MRI inflammation. In contrast, in other arthritis patients and in asymptomatic volunteers, a higher BMI was associated with higher MRI inflammation scores (respectively β = 1.082, *p* < 0.001 and β = 1.029, *p* = 0.040; Fig. 1). When BMI was categorized into three groups (low-normal weight, overweight, and obese), similar results were obtained. Obese RA patients had significantly lower MRI inflammation scores (median = 10.0, IQR = 6.5–18.0) compared with low-normal weight RA patients (median = 19.5, IQR = 7.5–29.0, *p* = 0.005) and overweight RA patients (median = 14.5, IQR = 7.0–28.0, *p* = 0.046). Within the group of other early arthritis patients, obese patients had higher total MRI inflammation scores (median = 8.5, IQR = 5.0–13.5) compared with patients with a low-normal weight (median = 2.5, IQR = 0.5–9.5, *p* = 0.002). Similarly, overweight was also associated with higher total MRI inflammation scores (median = 8.0, IQR = 3.5–22.0, *p* < 0.001). Within asymptomatic volunteers a tendency towards higher inflammation scores was seen in obese persons (median = 3.5, IQR = 1.0–5.5) compared with low-normal weight persons (median = 1.5, IQR = 0.5–3.5, *p* = 0.064) and overweight persons (median = 2.0, IQR = 1.0–4.5, *p* = 0.24; Fig. 1).

Thereafter, the association between BMI (measured continuously) and MRI-detected inflammation was adjusted for age and gender, also showing that a higher BMI was associated with higher inflammation scores in other early arthritis patients (β = 1.036, *p* = 0.054) and asymptomatic volunteers (β = 1.022, *p* = 0.040) but with lower inflammation scores in RA patients (β = 0.96, *p* = 0.005; Table 2 (for nonback-transformed β values, see Additional file 3)). After additional adjustments for CRP and anti-citrullinated protein antibody (ACPA) the results remained similar (β = 0.96, *p* = 0.003 for RA patients and β = 1.039, *p* = 0.043 for other early arthritis patients).

**Table 2:** Association of BMI with MRI-detected inflammation in patients with RA, early arthritis patients with other arthritides, and asymptomatic volunteers

	Rheumatoid arthritis (*n* = 195)		Other arthritides (*n* = 159)		Asymptomatic volunteers (*n* = 193)	
	β (95 % CI)	*p* value	β (95 % CI)	*p* value	β (95 % CI)	*p* value
Univariable						
BMI	0.97 (0.94–1.00)	0.024	1.082 (1.041–1.13)	<0.001	1.029 (1.001–1.057)	0.040
Multivariable						
Model 1						
BMI	0.96 (0.94–0.99)	0.005	1.036 (1.00–1.075)	0.054	1.022 (1.001–1.044)	0.040
Age	1.025 (1.017–1.033)	< 0.001	1.033 (1.024–1.041)	< 0.001	1.031 (1.025–1.036)	< 0.001
Gender	1.13 (0.89–1.44)	0.30	0.88 (0.67–1.15)	0.34	1.010 (0.84–1.21)	0.92
Model 2						
BMI	0.96 (0.94–0.99)	0.003	1.039 (1.001–1.078)	0.043	NA	
Age	1.022 (1.014–1.030)	< 0.001	1.030 (1.021–1.039)	< 0.001	NA	
Gender	1.12 (0.89–1.41)	0.35	0.93 (0.71–1.23)	0.62	NA	
CRP	1.007 (1.003–1.012)	0.001	1.002 (0.998–1.006)	0.29	NA	
ACPA positivity	0.96 (0.77–1.20)	0.72	1.55 (0.75–3.20)	0.24	NA	

Total inflammation scores were log-transformed for regressions. Regression coefficients presented are back-transformed (10^β^ and 10^95 % CI^). Therefore, the effect size (β) can be interpreted as the fold increase in MRI-detected inflammation per point increase in BMI. Thus, an effect size of < 1 means a decrease in MRI-detected inflammation per unit increase in BMI and an effect size of > 1 means an increase in MRI-detected inflammation per unit increase in BMI. The raw beta coefficients are presented in Additional file 3

*BMI* body mass index, *MRI* magnetic resonance imaging, *RA* rheumatoid arthritis, *NA* not assessed, *CRP* C-reactive protein, *ACPA* anti-citrullinated protein antibody

The group of early arthritis patients with diagnoses other than RA was divided into the following six subgroups: inflammatory osteoarthritis (*n* = 38), spondyloarthritis with peripheral arthritis and psoriatic arthritis (*n* = 40), systemic lupus erythematosus/mixed connective tissue disease and other systemic disease (*n* = 20), reactive arthritis and lyme arthritis (*n* = 17), gout and pseudogout (*n* = 19), and other diseases (*n* = 25). These subgroups were studied to assess whether the association was more pronounced in a particular disease group. However, the directionality of the effect was similar in all subgroups (Additional file 4).

### BMI and different types of MRI inflammation

The total MRI inflammation score is composed of the synovitis, tenosynovitis, and BME scores. To assess whether synovitis, BME, and tenosynovitis have different associations with BMI, these types of inflammation were assessed separately. The synovitis score showed a negative association with BMI in RA patients (β = 0.98, *p* = 0.047) and a positive association in other arthritis patients and in asymptomatic volunteers (β = 1.084, *p* < 0.001 and β = 1.031, *p* = 0.006 respectively). A higher BMI was associated with lower BME in RA patients (β = 0.95, *p* = 0.002). In other arthritis patients and in asymptomatic volunteers, BMI was not associated with BME scores (respectively β = 1.021, *p* = 0.24 and β = 1.003, *p* = 0.79). Within other arthritis patients and in asymptomatic volunteers there was a positive association between BMI and the tenosynovitis score (β = 1.054, *p* = 0.003 and β = 1.021, *p* = 0.003 respectively), whereas BMI was not associated with tenosynovitis in RA (β = 0.98, *p* = 0.21; Additional file 5).

### Further analyses in BMI and BME

**Fig. 2:**
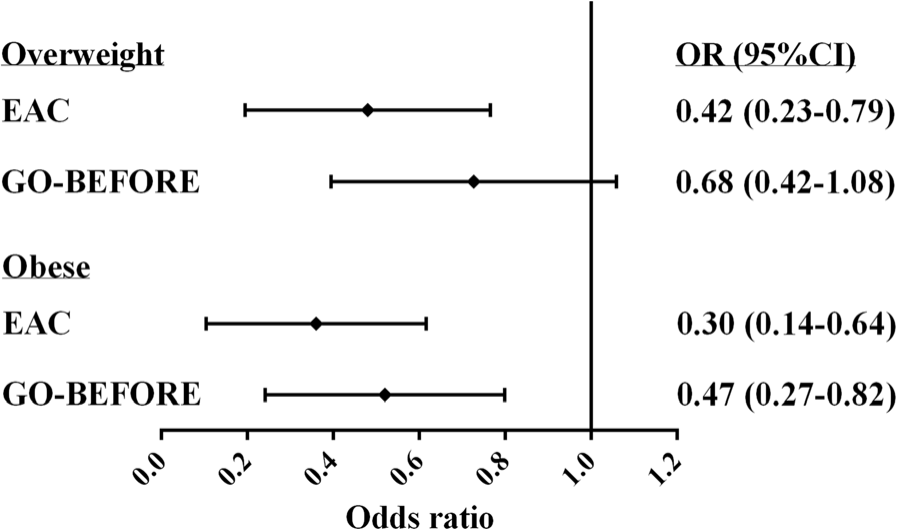
Association of overweight and obesity with BME compared with low-normal weight in RA patients included in the EAC cohort and the GO-BEFORE trial [3]. BMI was categorized into three groups: low-normal weight (< 25 kg/m^2^), overweight (≥ 25 to < 30 kg/m^2^), and obese (≥ 30 kg/m^2^). Odds ratios (ORs) were calculated with low/normal weight as the reference category. *EAC* Early Arthritis Clinic

Recently, an inverse association between BMI and BME was shown in RA patients who were treated in a trial [3]. The median BME scores of patients included in this trial and who were low-normal weight, overweight, and obese patients were respectively 9 (IQR 2.5–19), 6.3 (IQR 2.5–13), and 4.8 (IQR 1.5–9.8) [3]. We wished to compare these recent results with our findings obtained in an unselected set of RA patients at the time of disease onset. The median scores observed in our cohort showed a similar tendency but were lower; the median scores in the three groups were respectively 5.0 (IQR 2.0–11.0), 3.0 (IQR 1.0–9.0), and 2.0 (IQR 1.0–4.5). The trial data showed that, after adjusting for race, ACPA, disease duration, DAS, age, and sex, overweight patients had an odds ratio (OR) of 0.68 (95 % CI 0.42–1.08, *p* = 0.1) and obese patients an OR of 0.47 (95 % CI 0.27–0.82, *p* = 0.008) for being in a higher BME quintile (Fig. 2). We performed the same analyses without adjusting for race because our study population consisted of 96 % Caucasians, and without disease duration because all of our early arthritis patients were evaluated at their first presentation to the rheumatologic outpatient clinics. In our data, overweight patients had an OR of 0.42 (95 % CI 0.23–0.79, *p* = 0.007) and obese patients had an OR of 0.30 (95 % CI 0.14–0.64, *p* = 0.002) compared with low-normal weight patients (Fig. 2). Analyses of both data sets thus showed an inverse association between BMI and BME scores.

### BMI and regular measures of inflammation

The association of BMI with regular measures of inflammation was assessed in RA patients and other arthritis patients. In RA patients the median swollen joint count (SJC) in low-normal weight patients was 5 (IQR = 3–10), in overweight patients the median was 6 (IQR = 2–10), and in obese patients the median was 8 (IQR = 3–10; *p* = 0.69). The median CRP levels in low-normal weight, overweight, and obese RA patients were respectively 7 mg/L (IQR = 3–23 mg/L), 12 mg/L (IQR = 4–23 mg/L), and 10 mg/L (IQR = 4–24 mg/L; *p* = 0.39). In other arthritis patients the median of SJC in low-normal weight, overweight, and obese patients were respectively 3 (IQR = 1–5), 3 (IQR = 2–5), and 4 (IQR = 3–7; *p* = 0.074). Lastly, the median CRP level in the three groups were respectively 4 mg/L (IQR = 3–18 mg/L), 4 mg/L (IQR = 3–12 mg/L), and 5 mg/L (IQR = 3–13 mg/L; *p* = 0.65). Therefore, in RA patients and in other arthritis patients, BMI was neither associated with swollen SJC nor with CRP levels.

### BMI and ACPA in RA patients

The influence of BMI on MRI-detected inflammation in ACPA-positive or ACPA-negative RA patients was assessed separately. Although the subgroups became small (*n* = 107 and *n* = 88 respectively), the effect size of the association between BMI and MRI-detected inflammation remained unchanged (respectively β = 0.97, *p* = 0.071 and β = 0.97, *p* = 0.12 for ACPA-positive RA and ACPA-negative RA in the univariable analyses and respectively β = 0.97, *p* = 0.097 and β = 0.96, *p* = 0.011 for the analyses adjusted for age and gender).

Furthermore, the association between ACPA level and BMI was assessed with the Spearman rank test in all RA patients. ACPA levels were not correlated with BMI (ρ = −0.33, *p* = 0.65) and did not differ between the three BMI categories (*p* = 0.38; Additional file 6).

## Discussion

An increased BMI is associated with higher inflammatory markers in the general population [1], and a higher risk for RA development [2]. A high BMI within RA, however, is associated with less severe radiographic joint damage [3–6]. Because joint destruction is the result of persistent inflammation, the present cross-sectional study assessed the association between BMI and MRI-detected inflammation and showed that RA patients with a high BMI had less MRI-detected inflammation. More specifically, patients had less severe synovitis and BME. This inverse association was not observed in early arthritis patients with other inflammatory diagnoses and in asymptomatic volunteers. This suggests that the inverse association between BMI and local joint inflammation is confined to RA and may explain the previously reported observation of less severe radiographic progression in obese RA patients.

The mechanism underlying this inverse association is unknown. It can be speculated that adipocytokines play a role. It could be that the composition of the adipocytokines is different between various diseases; for example, the balance between low molecular weight versus high molecular weight adiponectin might be different. Another possibility is that the interaction between adipocytokines and immune cells is different within RA compared with other diseases. However, we have no data to support these speculations and further studies are needed to unravel the biologic mechanism underlying our observation.

To the best of our knowledge RA is the only disease in which obese patients have less severe inflammation and progression. Also, the effect of obesity for RA is two-fold. Despite the association with less severe MRI-detected inflammation and less severe radiographic progression, obesity has been associated with a higher risk for developing RA and a lower risk for reaching persistent remission [2, 12]. Furthermore, a lower chance to achieve a low disease activity has also been observed in RA patients that use synthetic DMARDs and biological DMARDs [13–15]. Of note, when evaluating the components of the disease activity score, the effect was only present for subjective measures (tender joint count and patient global assessment) and not for objective measures of inflammation (CRP, erythrocyte sedimentation rate, and swollen joint count) [13].

Also in the present study we observed no association between BMI and either the CRP levels or the number of swollen joints. This illustrates that local inflammation is different from systemic inflammation and also underlines that MRI is a more sensitive method to detect local inflammation than physical examination of joints. Apparently the less severe radiographic progression in RA is paralleled by less severe local inflammation, which is detected when local inflammation is measured using a sensitive method. As such, the results of the present study suggest that MRI is not only valuable as an outcome measure in clinical trials but that MRI studies may also help to increase our pathophysiological understanding of RA.

In line with recommendations of the ESSR [16], BME was evaluated on T1 Gd, which is different from the RAMRIS methodology using T2. Our scan protocol omitted T2 because previous studies have shown that these sequences perform equally well in the depiction of BME [17, 18] and the T1 Gd sequence allows a shorter imaging time for the patients. The present finding of similar effects of BMI in BME as observed in two different studies in which BME was assessed on different sequences (Baker et al. [3] used short tau inversion recovery (or T2 precontrast) sequences) support the notion that the findings are not influenced by the sequence used to depict BME.

This study has limitations. The BMI was used as an estimate of the adipose tissue, but differences in BMI are not only caused by differences in adipose tissue but also by differences in, for instance, muscle mass. There are methods that could make more accurate estimations in this respect, such as waist circumference, bioelectrical impedance, or computed tomography. Another important limitation is that long-term follow-up was not yet available for the RA patients who had undergone MRI. Therefore we could not assess whether our findings at disease presentation might explain the association of BMI with less severe radiographic joint progression. In addition we could not determine the association of BMI with other disease outcomes, such as persistent remission.

## Conclusions

The association between BMI and MRI-detected inflammation differs in patients with RA compared with patients with other inflammatory diagnoses and with asymptomatic controls. Within RA a higher BMI is associated with less severe MRI-detected inflammation, and this may explain the finding that obese RA patients have less severe radiographic progression.

## Additional files

Additional file 1: presents the detailed MRI scan protocol. (DOCX 24 kb)
Additional file 2: is a table presenting the ICC for total inflammation score for 30 arthritis patients scored by all four readers. (DOCX 14 kb)
Additional file 3: is a table presenting raw beta coefficients for the association of BMI with MRI-detected inflammation in patients with RA, in early arthritis patients with other arthritides, and in asymptomatic volunteers. (DOCX 18 kb)
Additional file 4: is a figure showing correlations of BMI with MRI-detected inflammation in inflammatory osteoarthritis, spondyloarthritis with peripheral arthritis and psoriatic arthritis, systemic lupus erythematosus, mixed connective tissue disease and other systemic diseases, reactive arthritis and lyme arthritis, gout and pseudogout, and other diseases. (DOCX 319 kb)
Additional file 5: is a figure showing associations of BMI with synovitis, BME, and tenosynovitis in patients with early RA, early arthritis patients with other diagnoses, and asymptomatic volunteers. (DOCX 120 kb)
Additional file 6: is a figure showing the association between BMI (both when presented on a continuous scale or categorized) and ACPA titers in early RA patients. (DOCX 218 kb)

## Abbreviations

ACPA: Anti-citrullinated protein antibody after intravenous contrast administration
BME: Bone marrow edema
BMI: Body mass index
CRP: C-reactive protein
EAC: Early Arthritis Clinic
ICC: Intraclass correlation coefficients
MCP: Metacarpophalangeal
MRI: Magnetic resonance imaging
MTP: Metatarsophalangeal
RA: Rheumatoid arthritis
T1: T1 fast-spin echo
T1Gd: T1 fast-spin echo with fat saturation
T2: T2-weighted fat saturated sequence
WHO: World Health Organization
