# Supplementary material for: Body mass index and extent of MRI-detected inflammation: opposite effects in rheumatoid arthritis versus other arthritides and asymptomatic persons
Source: Arthritis Res Ther. 2016 Oct 22;18:245. doi: 10.1186/s13075-016-1146-3 (PMC5075146; doi:10.1186/s13075-016-1146-3)
Supplement: Additional file 1: — presents the detailed MRI scan protocol. (DOCX 24 kb) [file 13075_2016_1146_MOESM1_ESM.docx]

**Detailed MR-scan protocol**

MR imaging was performed on a MSK-extreme 1.5T extremity MR imaging system (GE, Wisconsin, USA) using a 145mm coil for the foot and a 100mm coil for the hand. The patient was positioned in a chair beside the scanner, with the hand or foot fixed in the coil with cushions.

In the hand (MCP2-5 and wrist) the following sequence was acquired before contrast administration: T1-weighted fast spin-echo (FSE) sequence in the coronal plane (repetition time (TR) 575 ms, echo time (TE) 11.2 ms, acquisition matrix 388×288, echo train length (ETL) 2). After intravenous injection of gadolinium contrast (gadoteric acid, Guerbet, Paris, France, standard dose of 0.1 mmol/kg) the following sequences were obtained: T1-weighted FSE sequence with frequency selective fat saturation (fatsat) in the coronal plane (TR/TE 700/9.7ms, acquisition matrix 364×224, ETL 2), T1-weighted FSE sequence with frequency selective fat saturation in the axial plane (wrist: TR/TE 540/7.7 ms; acquisition matrix 320x192; ETL 2 and MCP-joints: TR/TE 570/7.7 ms; acquisition matrix 320x192; ETL 2).

The obtained sequences of the forefoot (MTP1-5 joints) were for the first 371 patients before contrast administration: T1-weighted FSE sequence in the axial plane (TR/TE 650/17ms; acquisition matrix 388x288, ETL 2); and T2-weighted FSE fatsat sequence in the axial plane (TR/TE 3000/61.8; acquisition matrix 300x224, ETL 7). Imaging of the foot was initially limited to pre-contrast axial sequences. For the later 218 patients post-contrast sequences were included: T1-weighted FSE fatsat sequence in the axial plane (TR/TE 700/9.5ms; acquisition matrix 364x224, ETL 2) and: T1-weighted FSE fatsat sequence in the coronal plane (perpendicular to the axis of the metatarsals) (TR/TE 540/7.5ms; acquisition matrix 320x192, ETL 2).

Field-of-view was 100mm for the hand and 140mm for the foot. Coronal sequences of the hand had 18 slices with a slice thickness of 2mm and a slice gap of 0.2mm. Coronal sequences of the foot had 20 slices with a slice thickness of 3mm and a slice gap of 0.3mm. All axial sequences had a slice thickness of 3mm and a slice gap of 0.3mm with 20 slices for the wrist, 16 for the metacarpophalangeal-joints and 14 for the foot. According to the RAMRIS-method, T2-weighted fat suppressed sequences, or when this sequence is not available a short tau inversion recovery (STIR) sequence, should be used to assess BME. Previously, three studies have demonstrated that a contrast enhanced T1-weigthed fat suppressed (T1gd) sequence has a strong correlation with T2-weighted fat suppressed sequences, (1–4) additionally in the recommendations of the European Society of musculoSkeletal Radiology (ESSR) it is stated that T1gd could also depict BME. T1gd, T2 and STIR showed similar contrast in images with BME. The reason for this is the increased perfusion and interstitial leakage (provided there is no increased interstitial pressure) and increased water content. Apart from enhancement, the increased water content is also used to create increased signal in T1-weighted fat sat sequences, even without the use of contrast agents. As opposed to for instance the calibrated Hounsfield unit on CT, the MR signal intensities are relative values. By taking away the high signal intensity of fat in T1-weighted images, the signal intensity scale is changed completely and tissue with some water content will move up the scale and low signal intensity will become relatively high signal intensity. Therefore high signal intensity on T1gd images is a result of both water content and enhancement. Because of this reasoning, because three studies showed that both methods performed equally, also in patients without inflammatory diseases such as bone bruises, intraosseous ganglions, bone infarcts and even nonspecific cases, and because of the ESSR recommendation, the T1gd sequence was used to limit the scan time (these were already made to assess synovitis and tenosynovitis) and it has a higher signal to noise ratio. (1–4)

**MRI scoring**

All bones and joints were scored semi-quantitatively in line with the validated RA MRI scoring system (RAMRIS, also applied at the MTP joints). Tenosynovitis was scored according to the method described by Havaardsholm (also applied at the flexor and extensor tendons at the 2^nd^-5^th^ MCP-joints). Erosions were scored on a 0-10 scale based on the percentage of eroded bone (0, no eroded bone, 1 >0-10%, 2 11-20% etc.), BME was scored on a 0-3 scale based on the affected volume of the bone (no BME, >0-33%, >33-66%, >66%), the synovitis score (range 0-3) was scored based on the volume of enhancing tissue in the synovial compartment (none, mild, moderate, severe) and the tenosynovitis-score (ranged 0-3) was based on the thickness of peritendinous effusion or synovial proliferation with contrast enhancement (normal, <2mm, 2-5mm, >5mm). (5,6) The scores of all joints were summed and the total BME, synovitis and tenosynovitis scored were summed as well, yielding the total MRI inflammation score.

References

1. Stomp W, Krabben A, Heijde D van der, Huizinga TWJ, Bloem JL, Mil AHM van der H, et al. Aiming for a shorter rheumatoid arthritis MRI protocol: can contrast-enhanced MRI replace T2 for the detection of bone marrow oedema? *Eur Radiol* 2014;24:2614–2622.

2. Mayerhoefer ME, Breitenseher MJ, Kramer J, Aigner N, Norden C, Hofmann S. STIR vs. T1-weighted fat-suppressed gadolinium-enhanced MRI of bone marrow edema of the knee: Computer-assisted quantitative comparison and influence of injected contrast media volume and acquisition parameters. *J Magn Reson Imaging* 2005;22:788–793.

3. Schmid MR, Hodler J, Vienne P, Binkert CA, Zanetti M. Bone Marrow Abnormalities of Foot and Ankle: STIR versus T1-weighted Contrast-enhanced Fat-suppressed Spin-Echo MR Imaging. *Radiology* 2002;224:463–469.

4. Sudoł-Szopińska I, Jurik AG, Eshed I, Lennart J, Grainger A, Østergaard M, et al. Recommendations of the ESSR Arthritis Subcommittee for the Use of Magnetic Resonance Imaging in Musculoskeletal Rheumatic Diseases. *Semin Musculoskelet Radiol* 2015;19:396–411.

5. Haavardsholm EA, Ostergaard M, Ejbjerg BJ, Kvan NP, Kvien TK. Introduction of a novel magnetic resonance imaging tenosynovitis score for rheumatoid arthritis: reliability in a multireader longitudinal study. *Ann Rheum Dis* 2007;66:1216–1220.

6. Østergaard M, Peterfy C, Conaghan P, McQueen F, Bird P, Ejbjerg B, et al. OMERACT Rheumatoid Arthritis Magnetic Resonance Imaging Studies. Core set of MRI acquisitions, joint pathology definitions, and the OMERACT RA-MRI scoring system. *J Rheumatol* 2003;30:1385–1386.
