# Supplementary material for: Body mass index and extent of MRI-detected inflammation: opposite effects in rheumatoid arthritis versus other arthritides and asymptomatic persons
Source: Arthritis Res Ther. 2016 Oct 22;18:245. doi: 10.1186/s13075-016-1146-3 (PMC5075146; doi:10.1186/s13075-016-1146-3)
Supplement: Additional file 2: — is a table presenting the ICC for total inflammation score for 30 arthritis patients scored by all four readers. (DOCX 14 kb) [file 13075_2016_1146_MOESM2_ESM.docx]

**Additional file 2.** Intraclass correlation coefficients for total inflammation score for 30 arthritis patients scored by all 4 readers.

|  | ECN | WN | LM | HVS |
| --- | --- | --- | --- | --- |
| ECN | x | 0.96 | 0.96 | 0.97 |
| WN | 0.96 | x | 0.92 | 0.92 |
| LM | 0.96 | 0.92 | x | 0.97 |
| HVS | 0.97 | 0.92 | 0.97 | x |
