# Supplementary material for: Body mass index and extent of MRI-detected inflammation: opposite effects in rheumatoid arthritis versus other arthritides and asymptomatic persons
Source: Arthritis Res Ther. 2016 Oct 22;18:245. doi: 10.1186/s13075-016-1146-3 (PMC5075146; doi:10.1186/s13075-016-1146-3)
Supplement: Additional file 3: — is a table presenting raw beta coefficients for the association of BMI with MRI-detected inflammation in patients with RA, in early arthritis patients with other arthritides, and in asymptomatic volunteers. (DOCX 18 kb) [file 13075_2016_1146_MOESM3_ESM.docx]

**Additional file 3.** Raw beta coefficients of the association of BMI with MRI-detected inflammation in patients with RA, early arthritis patients with other arthritides and asymptomatic volunteers.

|  | Rheumatoid arthritis (n=195) | | Other arthritides (n=159) | | Symptom-free (n=193) | |
| --- | --- | --- | --- | --- | --- | --- |
|  | β (95%CI) | p | β (95%CI) | p | β (95%CI) | p |
| Univariable |  |  |  |  |  |  |
| BMI | -0.014 (-0.025;-0.002) | 0.024 | 0.034 (0.017;0.051) | <0.001 | 0.012 (0.001;0.024) | 0.040 |
| Multivariable |  |  |  |  |  |  |
| Model 1 |  |  |  |  |  |  |
| BMI | -0.016 (-0.027;-0.005) | 0.005 | 0.016 (0.000;0.031) | 0.054 | 0.009 (0.000;0.019) | 0.040 |
| Age | 0.011 (0.007;0.014) | <0.001 | 0.014 (0.010;0.017) | <0.001 | 0.013 (0.011;0.015) | <0.001 |
| Gender | 0.054 (-0.049;0.157) | 0.299 | -0.057 (-0.173;0.060) | 0.337 | 0.004 (-0.074;0.082) | 0.917 |
| Model 2 |  |  |  |  |  |  |
| BMI | -0.016 (-0.027;-0.006) | 0.003 | 0.017 (0.001;0.033) | 0.043 | NA |  |
| Age | 0.009 (0.006;0.013) | <0.001 | 0.013 (0.009;0.016) | <0.001 | NA |  |
| Gender | 0.048 (-0.052;0.149) | 0.345 | -0.030 (-0.149;0.090) | 0.623 | NA |  |
| CRP | 0.003 (0.001;0.005) | 0.001 | 0.001 (-0.001;0.003) | 0.294 | NA |  |
| ACPA | -0.018 (-0.116;0.081) | 0.720 | 0.190 (-0.125;0.506) | 0.235 | NA |  |

NA, not assessed
