# Supplementary material for: Body mass index and extent of MRI-detected inflammation: opposite effects in rheumatoid arthritis versus other arthritides and asymptomatic persons
Source: Arthritis Res Ther. 2016 Oct 22;18:245. doi: 10.1186/s13075-016-1146-3 (PMC5075146; doi:10.1186/s13075-016-1146-3)
Supplement: Additional file 4: — is a figure showing correlations of BMI with MRI-detected inflammation in inflammatory osteoarthritis, spondyloarthritis with peripheral arthritis and psoriatic arthritis, systemic lupus erythematosus, mixed connective tissue disease and other systemic diseases, reactive arthritis and lyme arthritis, gout and pseudogout, and other diseases. (DOCX 319 kb) [file 13075_2016_1146_MOESM4_ESM.docx]

**Additional file 4.** Correlations of BMI with MRI-detected inflammation in inflammatory osteoarthritis (A), spondyloarthritis with peripheral arthritis (SpA) & psoriatic arthritis (PsA) (B), systemic lupus erythematosus (SLE), mixed connective tissue disease (MCTD) & other systemic diseases (C), reactive arthritis & lyme arthritis (D), gout & pseudogout (E) and other diseases (F).

| 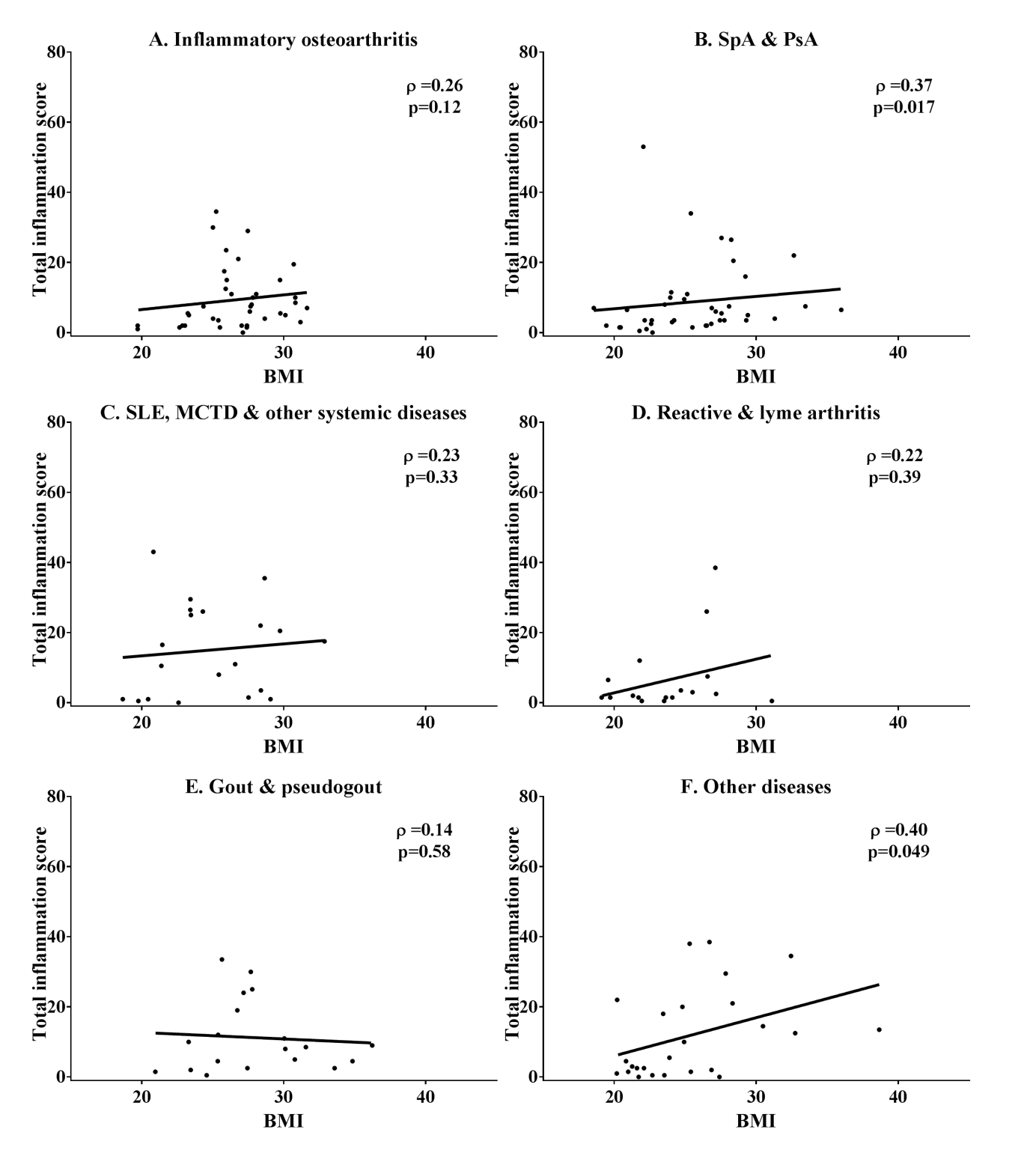 |
| --- |
|  |
