# Supplementary material for: Body mass index and extent of MRI-detected inflammation: opposite effects in rheumatoid arthritis versus other arthritides and asymptomatic persons
Source: Arthritis Res Ther. 2016 Oct 22;18:245. doi: 10.1186/s13075-016-1146-3 (PMC5075146; doi:10.1186/s13075-016-1146-3)
Supplement: Additional file 5: — is a figure showing associations of BMI with synovitis, BME, and tenosynovitis in patients with early RA, early arthritis patients with other diagnoses, and asymptomatic volunteers. (DOCX 120 kb) [file 13075_2016_1146_MOESM5_ESM.docx]

**Additional file 5.** Associations of BMI with synovitis (A,B,C), BME (D,E,F) and tenosynovitis (G,H,I) in patients with early RA (A,D,G), early arthritis patients with other diagnoses (B,E,H) and asymptomatic volunteers (C,F,I).

|  |
| --- |
| In RA-patients regression coefficients are 0.98 (95%CI 0.95;1.00) for synovitis, 0.95 (95%CI 0.93;0.98) for BME and 0.98 (95%CI 0.96;1.01) for tenosynovitis. In early arthritis patients with other diseases beta’s were 1.084 (95%CI 1.051;1.118) , 1.021 (95%CI 0.986;1.057) and 1.054 (95%CI 1.018;1.091) for respectively synovitis, BME and tenosynovitis. In asymptomatic volunteers they were respectively 1.031 (95%CI 1.009;1.053), 1.003 (95%CI 0.982;1.025) and 1.021 (95%CI 1.007;1.035). |
