# Supplementary material for: Body mass index and extent of MRI-detected inflammation: opposite effects in rheumatoid arthritis versus other arthritides and asymptomatic persons
Source: Arthritis Res Ther. 2016 Oct 22;18:245. doi: 10.1186/s13075-016-1146-3 (PMC5075146; doi:10.1186/s13075-016-1146-3)
Supplement: Additional file 6: — is a figure showing the association between BMI (both when presented on a continuous scale or categorized) and ACPA titers in early RA patients. (DOCX 218 kb) [file 13075_2016_1146_MOESM6_ESM.docx]

**Additional file 6.** The association between BMI (both when presented on a continuous scale or categorized) and ACPA titiers in early RA-patients.

| 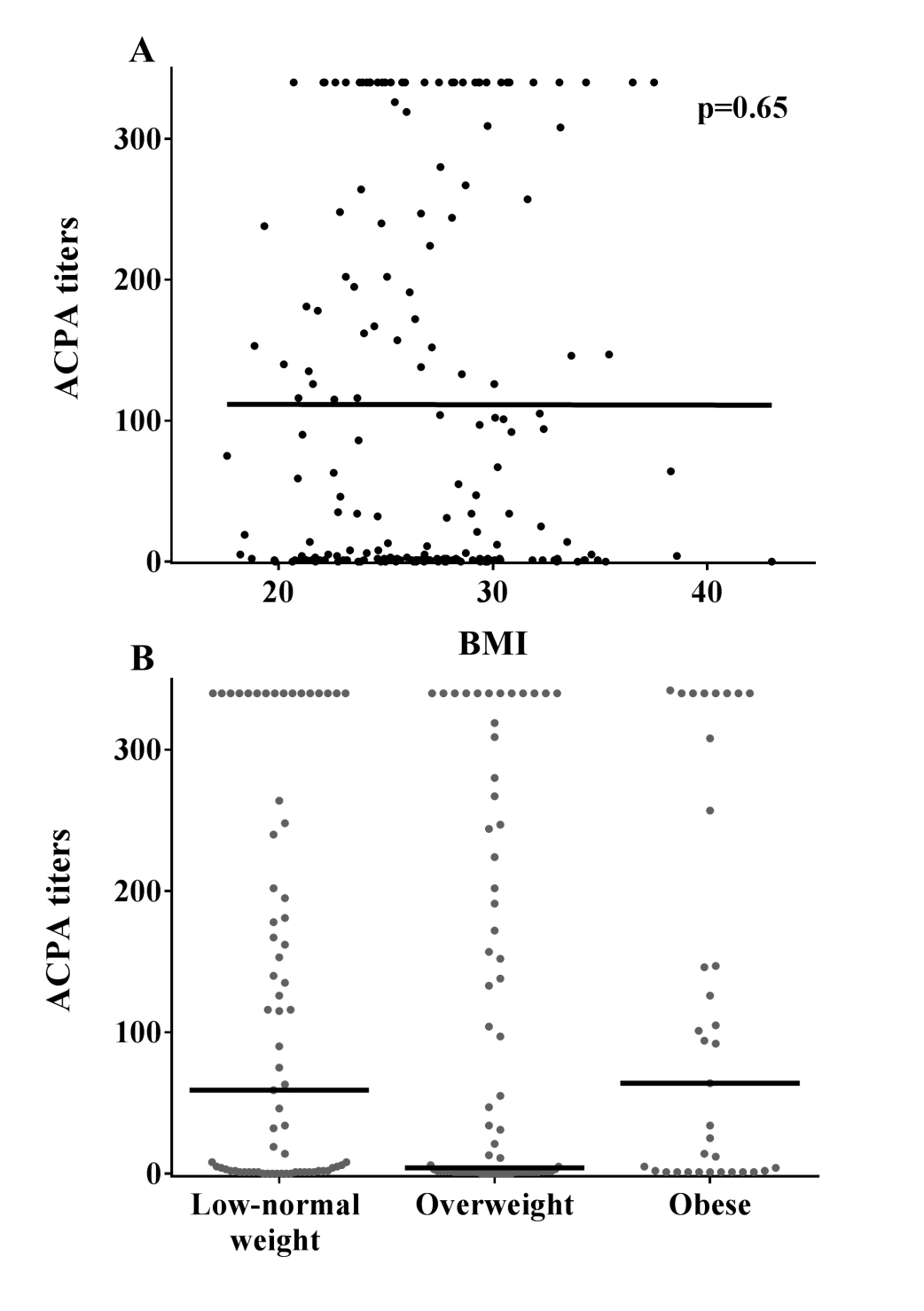 |
| --- |
| The spearman correlation between ACPA titers and BMI was -0.33 (p=0.65). The ACPA-titers were not different between the 3 BMI groups (p=0.38). Due to the detection limit which was set at 340 U/ml, many patients have the same ACPA level of 340 U/ml. |
